# The Unspeakable Nature of Death & Dying During Childhood: A Silenced Phenomenon in Pediatric Care

**DOI:** 10.1177/00302228211067034

**Published:** 2022-01-14

**Authors:** Sydney Campbell, Fiona J. Moola, Jennifer L. Gibson, Jeremy Petch, Avram Denburg

**Affiliations:** 1 Institute of Health Policy, Management and Evaluation, Dalla Lana School of Public Health, 7938 University of Toronto, Toronto, ON, Canada; 2 Joint Centre for Bioethics, Dalla Lana School of Public Health, 120913 University of Toronto, Toronto, ON, Canada; 3 School of Early Childhood Studies, 7984 Ryerson University, Toronto, ON, Canada; 4 37205 Holland Bloorview Kids Rehabilitation Hospital, Toronto, ON, Canada; 5 Dalla Lana School of Public Health, 274071 University of Toronto, Toronto, ON, Canada; 6 Rehabilitation Sciences Institute, 7938 The University of Toronto, Toronto, ON, Canada; 7 Division of Clinical Public Health, Dalla Lana School of Public Health, 274071 University of Toronto, Toronto, ON, Canada; 8 Centre for Data Science and Digital Health, 3708 Hamilton Health Science Centre, Hamilton, ON, Canada; 9 Division of Cardiology, 3710 McMaster University, Hamilton, ON, Canada; 10 33493 Population Health Research Institute, Hamilton, ON, Canada; 11 Division of Haematology and Oncology, 7979 The Hospital for Sick Children, Toronto, ON, Canada; 12 Department of Pediatrics, Faculty of Medicine, 12366 University of Toronto, Toronto, ON, Canada

**Keywords:** children, youth, attitudes toward death and dying, silence, euthanasia

## Abstract

In pediatric settings, the concept of hope is frequently positioned as a fundamental aspect of care and at odds with the possibility and proximity of death. This arguably fosters silence about death and dying in childhood despite evidence indicating the benefits of open communication at the end of life. In this paper, we describe the unspeakable nature of death and dying in childhood, including its conceptual and clinical causes and dimensions, its persistence, and the associated challenges for children and youth facing critical illnesses, their families, and society. We explore how the tension between hope and death can be reframed and apply our analysis to the context of medical assistance in dying for mature minors in Canada. Considering the lack of related literature, this paper offers initial reflections to form a framework for the unspeakable nature of death and dying in childhood and to advance the crucial need for research.

## Introduction

*“Life is for the living. Death is for the dead. Let life be like music. And death a note* ***unsaid****.”* ― Langston Hughes

Experiencing the death of a loved one is an undeniably profound loss for which there may be no words to express its sorrow, but the death of a child is often “unspeakable.” Many believe distress related to the death of a child is different, a more pronounced stressor, because the death of a child “betrays that assumption of a natural order in which children outlive their parents” (Carr, 2016, pp. 277–278; Hazzard et al., 1992) and disrupts the vision of our lives (Riches & Dawson, 1996). As such, the unspeakable nature of childhood death is a reflection of and response to that trauma which occurs before, during, and after moments of a child’s death.

In some high-income countries, public health measures and advances in modern medicine have led to significant improvements in the overall health of children and a precipitous decline in infant and child mortality compared to 100 years ago. In fact, with the advances of modern medicine, a relatively small percentage of a country’s yearly death statistics pertain specifically to children.^1^ In Canada, particularly, Statistics Canada data has shown that the average number of young people aged 19 and under who died between 2015 and 2019 was approximately 3,065, while the average overall mortality rate during this period was approximately 275,205. As such, childhood deaths make up just over 1.1% of Canada’s yearly mortality rate (Statistics Canada, 2020). Indeed, as a result of advances in medicine and technology, a growing number of children with diseases that were once considered fatal are now living to adulthood (Moola, 2012). However, death in childhood is still present and the relatively small population still represents real children and youth (i.e., young people) and their families that are impacted every day. While philosophers, clinicians, and policymakers discuss and debate the definition of a “good death,” the experience of dying for young people and their families is often described as too challenging to speak about (Bates & Kearney, 2015; Hooghe et al., 2018) and the result is that childhood death and dying continues to be a silenced social, cultural, psychological, and spiritual phenomenon, not unlike “a note unsaid” written by Langston Hughes in the 1930s (Hughes, 2001).

As a research team located in Toronto, Canada, the unspeakable nature of death in childhood emerged for us both in anecdotes shared by clinicians and through a literature review conducted as part of a larger study on medical assistance in dying (MAID) (“euthanasia”) and mature minors.^2^ Through preliminary work related to this project, it has been made obvious that a large gap exists in the literature related to sociological and theoretical explorations on the intersection of childhood and death. This gap is concerning because although children are not exempt from the possibility that conversations about death and dying can be meaningful, the unspeakable nature of childhood death imposes a reality whereby children become further silenced. The silence associated with childhood death can also lead to fear of death for family and caregivers and a perpetuation of silence and stigma associated with thoughtful conversations, clinical interactions, and research pertaining to childhood death. This is despite evidence indicating that discussions about death with children facing critical illnesses are rarely regretted by parents after a child’s death, though avoidance of these topics can be (Kreicbergs et al., 2004).

In this paper, we provide an introduction to the unspeakable nature of death in the context of childhood, consider the antithetical nature of hope and death—along with childhood and death—that perpetuate this silence, and highlight the concerns associated with these observations. We also connect our analysis to the case of MAID for mature minors in Canada to consider how dialogue on MAID for mature minors can be used as a bridge to traverse the tension between hope and death. We draw on our own collective and cumulative expertise and experiences, as a: (a) PhD candidate conducting research related to end-of-life care and MAID policies linked to young people, (b) professor, research scientist, and psychotherapist under supervision who has been extensively trained in using qualitative and arts-based methods to explore experiences of childhood disability, (c) philosopher and health policy ethics scholar, (d) scientist and executive working at the intersection of technology and health care delivery, and (e) pediatrician who cares for children with complex, chronic diseases and scientist focused on the ethics of health policy decision-making. We conclude with reflections on ways forward. Of note, our reflections on death are largely based in the white, Euro-Centric, and colonial medical and education systems in which we have been educated. We recognize the grave limitations of working from within this frame and, in the years to come, we hope to be enriched by learnings and teachings on childhood death from other cultures and racial groups. We also recognize that our geographic boundaries—within a North American context—may limit the applicability of this conversation for other jurisdictions, but considering the wide gaps on the nature of childhood death and dying, this paper offers a starting point for continual dialogue. In what follows, we start by providing insight on the important concepts and disciplinary lenses relevant to our work, before diving into our analysis.

## Key Concepts and Disciplinary Lenses

First, the ways in which critical illness, chronic illness, and medical complexity are understood is important to define for this paper as most of our discussion is focused on young people within these groups. Critical illnesses refer to clinical states in which a patient’s life is threatened and where a bodily system is at-risk of failure (Coulter Smith et al., 2014). Chronic illnesses refer to conditions that are characterized by their prolonged duration (often with an acute phase around the time of diagnosis), that they do not spontaneously resolve, and that are rarely entirely curative (Compas et al., 2012; Stanton et al., 2007). These two forms of illness are not mutually exclusive and literature is emerging related to pediatric chronic critical illnesses, which refers to illnesses that are prolonged, consist of an acute phase, and where patients rely on critical care interventions (e.g., those that are associated with multiorgan system dysfunction) (Shapiro et al., 2017). Finally, medical complexity refers to the situation where a person has at least one underlying chronic condition that involves frequent utilization of health resources, entails functional limitations that vary across time and space, and requires specialized health care needs that span therapeutic sectors (Cohen et al., 2011).

For this paper, principles related to sociology of childhood, otherwise referred to as “new social studies of childhood,” have facilitated and advanced our thinking (James & Prout, 1997; James et al., 1998). In this article, childhood is described as a social construction, whereby the meaning attributed to the notion of childhood differs across time, historical period, and space (James et al., 1998) and sociocultural contexts (Chen et al., 2017). Sociology of childhood scholars challenge dominant models of childhood advanced by developmental psychology (Piaget, 1936) by employing principles that position young people as human beings rather than human “becomings” (Qvortrup et al., 1994). Here, children are recognized as possessing competency, having opinions about how they want their lives to be governed (i.e., possess autonomy), and are agents with rights (James et al., 1998; Mayall, 2000; Salvadori, 2001). In this way, we are deeply critical of the dominance of developmentalism and the ways in which stage-like models of developmentalism inevitably leave some children—particularly those children with medical complexity and critical or chronic illnesses—behind in a way that often regards them as developmental failures.

Social constructionist interpretation of childhood is underpinned by a relational understanding of children, whereby young people influence *and* are influenced by their environment (James & Prout, 1997) and childhood, adolescence, and adulthood are understood through their interrelatedness (Chen et al., 2017). Accordingly, social discourses, institutions, relations, and norms also become influenced by how childhood and youth are understood. And while “maturation,” “aging,” or “development” is a naturally occurring phenomenon, there are various aspects of childhood (e.g., the age at which a child is recognized as an adult in their community) that are socially constructed and vary across time, space, and culture (Chen et al., 2017). The way society understands childhood as “an ontology [way of being] in its own right” (Jenks, 2005, p. 10; Moola & Norman, 2011) impacts the assumptions made about children’s capacities and this may differ depending on social context. The sociology of childhood also recognizes children as embodied health actors who possess detailed and intimate knowledge about their bodies and health (Mayall, 1998). The sociology of childhood thus recognizes children’s potential for or actual embodied and/or medical agency.

## The Unspeakable Nature of Death in Context of Childhood

In this paper, we define the **unspeakable nature of childhood death** as a phenomenon in which the entire end-of-life experience of a child or youth (also encompassing the time leading up to and following a child or youth’s death) is made silent for the young person themselves, but also for families, friends, and other members of the young person’s social circle, for clinicians, for researchers, and for society more broadly. Here, “silence” refers specifically to the lack of discussion, deliberation, and dialogue that occurs regarding the young person’s death and dying experience, although this purposefully does not include the ability to think about death by oneself or the necessary conversation between clinicians and family members regarding prognosis and end-of-life care options.

This silence arises *clinically*. For example, in the literature, and during some of our team’s research planning conversations with health care providers, health practitioners and researchers have shared their reflections on the hesitancy families reveal in clinical interactions when a child is toward the end of their life, making these conversations challenging (Freyer, 2004; Longbottom & Slaughter, 2018). Indeed, this perceived hesitancy often rests on the problematic assumption that death conversations are too troubling for children. There is also a *conceptual* silence, in that little research exists to elaborate on and specify how childhood death is theoretically understood. Further, there is little academic dialogue on how such silence is innately linked to death and dying in the context of childhood, resulting in a lack of advanced discussions on this topic, especially in modern times. There are likely two main reasons for this overarching clinical and conceptual climate of silence that we propose and elaborate on in what follows, namely: (1) The dominant ways that death and dying have previously been conceptualized do not fully encompass the experiences of death and dying during childhood. These dominant frameworks also support an assumption that conversations about death and dying are too challenging for children to tolerate or that such conversations will cause undue harm. And (2) the lower morbidity and mortality rates amongst children in a North American context in particular, due to medical advances, make conversations about death and dying in relation to childhood seem less relevant on the whole.

### The Undermining Role of Dominant Understandings of Death and Dying

Death has been conceptualized in numerous ways by philosophers of Western/Eurocentrism traditions and Eastern traditions. The Christian view of death, wherein death is seen as a personal transformation rather than a loss, is a prominent view that has influenced many understandings of death arising from a Western viewpoint (Collier & Haliburton, 2011, p. 215). There are also several medicalized ways of understanding death, based on anatomical terms, such as brain death (i.e., cessation of brain functioning constitutes the death of a person) or cardiorespiratory death (i.e., cessation of heart or lung functioning constitutes the death of a person) (Canadian Blood Services, 2012). Across most definitions of death, the assumption that death is always seen as “distasteful,” “bad,” or negative (Kübler-Ross, 1969, p. 2) prevails, leading to associated fear and/or anxiety (Kastenbaum & Costa, 1977; Stambrook & Parker, 1987). However, death is a truly complex phenomenon, both in an embodied and relational sense and—for some—with an ascribed spiritual component, that is challenging to universally define (Holland, 2017). This is equally true for the death of a young person, yet made more complex by the related silence associated with childhood death.

In Elisabeth Kübler-Ross’s highly cited, but deeply controversial, book entitled *On Death and Dying* (Kübler-Ross 1969), Kübler-Ross proposes the “Five Stage Model for Dying.” These stages are: denial, anger, bargaining, depression, and acceptance. Kübler-Ross has said that these stages are not linear, meaning a person can both enter and re-enter these stages in any order and that two stages can co-exist within a person’s response to and experience of dying (Corr, 2018). Yet, these dominant models do not always accurately map onto the way dying is experienced for young people who have critical illnesses and their families. As such, these models are based on an assumed dying adult, which Kübler-Ross has also previously highlighted (Kübler-Ross, 1983). More particularly, these adultist models have a somewhat idealistic picture of the process of dying in which knowing is ensured amongst all involved parties and “acceptance” is an *achievable* end state of coming to terms with an impending death.

While evidence has shown that children often do know when they are dying even when there is not an explicit conversation between parents/caregivers, clinicians, and children to disclose this information (Black, 1998; Bluebond-Langner, 1978; Spinetta, 1974; Waechter, 1971), the lack of *social* acceptance for parents (A. Matthews, 2019; Riches & Dawson, 1996) and society at large (Pomfret, 2015), though understandable, establishes an unspeakable nature to the phenomenon of childhood death. In return, this perpetual silence also undermines the possibility of achieving social acceptance—or even social *acknowledgement*—and undermines the possibility of *personal* acceptance on the part of the young person and their closest social circle, too. The dual role that silence plays in relation to childhood death and dying, both as a result of dominant interpretations of and fear regarding childhood death and as a mediator to perpetuate additional silence, undermines the possibility of meaningful conversations about childhood death and dying in both clinical and research spheres. Even in conversations about the death of a loved one, young people have recognized the way in which death and dying are unacknowledged and kept a “secret” in their lives. In these cases, where young people have desires and needs linked to the omnipresence of death in their lives, “death ambivalence” results as the product of tension from avoiding conversations about death with family, while simultaneously lacking opportunities to discuss death in a social context (Paul, 2019).

### The Potential Undermining Role of Long Life Expectancy

Aside from the various ways that childhood death and dying do not seem to be fully conceptually and clinically captured by the dominant models of death and dying, the otherwise positive advances in the treatment of childhood disease and illness may have also had a pivotal role in contributing to the silence associated with childhood death and dying narratives. Global research on childhood mortality rates, one key measure—along with infant mortality rates—of the health of a society (Centers for Disease Control and Prevention, 2021), has shown that over the last century there have been drastic reductions in the number of childhood deaths around the world. To be specific, in 1960, global child mortality was around 19% and in 2017 this rate dropped to 4% (Roser et al., 2019). Many believe this is a reflection of medical advances that have been seen throughout the twentieth and twenty-first centuries (Klass, 2020). Others report that the role of the social determinants of health (more specifically, an increase in the standard of living and a societal reorganization) have contributed to decreases in childhood mortality (Rydell, 1976); this has particular salience given the differential mortality rates amongst children from distinctive racial and/or ethnic groups or communities (MacDorman & Mathews, 2011). For example, Inuit, First Nations, and Métis communities face disproportionately higher infant mortality rates in Canada compared to non-Indigenous communities, at rates that are 3.9, 2.3, and 1.9 times the national average, respectively (Statistics Canada, 2019). In any case, today’s global childhood morality is the lowest it’s ever been (Roser et al., 2019), although efforts are underway to reduce this rate even further.

Yet, as the childhood morbidity and mortality rates are substantially reduced in many, though not all, countries around the world, we hypothesize that an unfortunate by-product is that the pertinence of theoretical clarity associated with the nuances of childhood death and dying are lost and silence is propagated in the realm of pediatric care. This is not to necessarily suggest the opposite is true: that childhood death and dying were easier to discuss when childhood mortality rates were higher^3^, or in jurisdictions currently facing higher rates of child mortality than Canada. Rather, our claim is that when the need to discuss a sensitive topic (like childhood death) can be avoided for the majority of a population, the status quo often becomes silence on that topic (Holahan et al., 2005). It is crucial to acknowledge that disparities in childhood survival exist across the globe, particularly disparities between high-and low-income countries. By corollary, exploration of the ways in which childhood death and dying are conceptualized or confronted differently, based on the diverse sociocultural and clinical contexts internationally and even in different regions of a single jurisdiction, is necessary for future research. And although it is true that 4% is a low percentage *relative to* the global child mortality rates of years past, this percentage still represents a large number of real young people that are being impacted. Therefore, conversations about death and dying with a child-lens are essential to consider rather than dismiss or abandon.

Not all sick children, however, have the luxury of expansive life-time and a normal life expectancy, indicating the value of open conversation on death and dying even in childhood. As illustrated by sociology of time scholars, time itself is an ideological construct that is rarely contemplated deeply unless threatened (Charmaz, 1991). In this way, time and its passage are deeply engrained and taken for granted truths that are rarely contemplated. Many adults, children, and even those with chronic illnesses, for example, are privileged with the benefit of knowing that they have a normal life expectancy and can anticipate a future time. For children with life-shortening illnesses, however, time and a long life expectancy are not necessarily something that can be counted on. In a paper by Moola and colleagues, exploring perceptions of physical activity by youth living with cystic fibrosis, this experience of time was highlighted by a 15-year-old female living with cystic fibrosis, who was keenly aware of her shortened life expectancy (Moola et al., 2012). She finds this very “hard,” leading to a sense of helplessness. Knowledge that she can no longer engage in physical activity because of her worsening condition is “depressing.” The following quote from the paper indicates this experience:

I also know that I am not going to live as long as everybody else, so that is hard. I feel like it is out of my control, I feel helpless, how I used to be able to do it (physical activity), and now I can’t. It is kind of depressing. It makes me think that it is a progressive disease, and it make me think that it is getting worse. . . it makes me worried. (p. 55)

## Challenging the Assumed Antithetical Nature of Hope and Death

Acknowledging the ways in which narratives about childhood death become overlooked and undermined leads us to question where the *foundational* rationale for the rejection of childhood mortality conversation lies. Based on interactions we’ve had through the processes of planning or being involved in distinctive research projects, particularly research related to MAID, or working in a clinical setting, we’ve noticed that there is a constant emphasis on hope’s inherent value and functionality in pediatric care settings. Emerging evidence also supports the belief in the value of hope for both children with underlying health conditions (Danielsen, 1995; Griggs & Walker, 2016; Hinds, 1988; Hinds et al., 1999; von Scheven et al., 2021) and their families (Leite et al., 2019). Broadly, hope is seen as foundational to how most societies envision childhood, whereby the possibility for future growth, exploration, development, achievements, and potential is linked to how childhood is often conceptualized. This also corresponds with narratives emerging at the end of the Second World War, whereby “children were highly symbolic of a hopeful future and successful reconstruction” (King et al., 2015). Relatedly, and more specific to pediatric healthcare, hope is seen as a “central” principle in pediatric medical practice (Seynnaeve et al., 2020) that is necessary to uphold and is fundamental to the ways parents, families (Kamihara et al., 2015; Szabat, 2020), and young people (Griggs & Walker, 2016; Hinds et al., 1999, 2000) cope with unpleasant news and/or critical diagnoses. Hope may also have an unspoken instrumental value in the context of critical illnesses, especially for children, as it provides motivation to persevere through the negative side effects of treatment for a chance at survival. While there are studies indicating that parents appreciate open and honest communication with providers, even as a means to support the family’s *realistic* hopes and/or to reimagine what “hope” can mean—aside from solely a curative focus (Kamihara et al., 2015)—there is a high degree of precedence for curative hope in the setting of pediatric care in particular.

This is where conversations about childhood death become challenging, as these are often perceived as being detrimental to the hope that is built by strong clinician-family relationships and throughout the child’s illness trajectory. In other words, when the potential for death and dying are mentioned in a pediatric setting, it is seen by some families and clinicians as a threat to a child and family’s sense of hope that was facilitated and built by the clinical team, the family themselves, their community/social circles, etc. In this way, the clinical environment often makes it challenging for hope and death to co-exist in the same space and continues to reproduce a problematic binary and polarity. Even if death is not a probable outcome for a pediatric patient, research-related discussions that are connected to death and dying discourses are similarly feared because they are perceived to collide with the hope that’s paramount to the ways in which clinicians and parents/caregivers provide treatment, care, and support to young people with critical and/or chronic illnesses. As such, it appears that hope is inherently tied to life, such that living a good life for a young person who is living with an illness requires hope’s presence. Hope is, therefore, seen as antithetical to discussions related to death and dying, whereby the co-existence of hope and childhood death/dying is seen as unlikely or even impossible. Considering that death is said to be unable to “constitute itself as an object of thought outside discourse” (Carpentier & Van Brussel, 2012, p. 99), it becomes essential to understand how the background—upon which both childhood and pediatric healthcare is understood and situated—contributes to and informs understandings of death and dying. As Carpentier and Van Brussel said, “although we should be careful not to reduce death to the way it is discursively interpreted, death remains loaded with meaning and we cannot detach it from the processes of social construction” (2012, p. 99).

This binary opposition between hope and death is also not entirely reflective of the reality that is experienced by those nearing the end of their life, particularly when employing a social constructionist view of childhood. As aforementioned, hope for a cure can be reformulated and re-envisioned into hope for other things, such as a high quality of life, a sense of normalcy, access to love and relationships, and reduced suffering (Friedel et al., 2018; Hill et al., 2014; Kamihara et al., 2015; Seynnaeve et al., 2020). Hope can also be reconstructed with a relational understanding of the child to recognize the value and importance of family, friends, clinicians, and the community to support and comfort a critically ill child at the end of life, and, likewise, to recognize the child’s role in supporting their social circle as well. In addition, children, parents/caregivers, and even clinicians can simultaneously hold hope for unrealistic expectations about prognosis, while acknowledging the realities of the child’s illness progression (Kamihara et al., 2015). Overall, sociology of childhood encourages families, clinicians, and researchers to acknowledge that hope can mean many things to different people and children should be supported, even and maybe especially at the end of life, to define what hope means to them and engage in conversations about death and dying if they wish to. Approaching the end of a young person’s life does not necessitate the cessation of care and it also does not necessitate the cessation of hope. Instead, death and dying in childhood can redirect the conversation to allow the embodied, emotional, and spiritual experiences of critical illnesses to be brought into a space that is focused on balancing reasonable expectations and realities with feelings of wistfulness and of fear. Yet, children are often holistically removed (some may say “spared”) from these conversations about death and dying, both in the clinic and in research, which allows the perceived binary opposition to continue undisputed. New research must challenge this discourse by engaging with young people, particularly those with lived experience of an illness, to understand how death and dying are understood and to confront the silence of childhood death and dying narratives. By perpetuating the status quo, children and their families continue to face many challenges and concerns.

## How Silence on Death and Dying Leads to Additional Concerns

Although our analysis thus far clarifies the ways in which conversations about death and dying remain unspeakable and silenced, some may question whether this is truly a problem. This perception often closely aligns with notions of childhood that emerge from developmental psychology, whereby children go through stages of cognitive growth and these stages impact their ability to understand, make sense of, and make meaning from their experiences and the experiences of others. Many proponents of development psychology also argue that death is understood differently and to varying degrees throughout childhood, depending on the age of a child (Carey, 1987; G. B. Matthews, 1989). As a result, silence may sometimes be seen as appropriate for developmental psychologists and for some, there is a strong emphasis on providing children with medical knowledge strictly based on their presumed or actual developmental stage (Brand et al., 2017). These scholars claim that when a child is in an early stage of cognitive development, they can only possess a “proto-concept of death” and “aren’t in the cognitive position to have the seriousness of their situation disclosed to them” (G. B. Matthews, 1989, p. 137). Thus, here the notion of silence in relation to childhood death arises, both in a clinical sense and a theoretical one, because conversations with children about death and its seriousness are assumed to not truly be absorbed by or tangible for children, especially those in pre-pubescent stages.

However, these theories overlook the challenges of so-called “universal childhood experiences”—challenges advanced by social constructionists—wherein cultural and social contexts lead to differences amongst children’s experiences, perspectives, interests, and capacities. In this way, encouraging silence is not necessarily an appropriate response for *all* children or for *all* developmental categories and universalized notions of child development and death may overlook precocious children who are capable of comprehending death and its consequences. These theories also overlook research indicating the ways in which children have already been impacted by silence related to their deteriorating condition, as described below, and negate the vast interpersonal world of very young children (Stern, 1985).

As previously indicated, sociology of childhood has emerged as a response to theories like development psychology that treat childhood as a means to the end of adulthood and that treat children as objects rather than agents. When it comes to understanding the capacities of a child to engage in conversations around death and dying, whether directly related to their own condition or that of a loved one, sociology of childhood encourages clinicians, parents, and child scholars to employ a case-by-case approach, whereby we neither assume the capacities of a child merely based on their age nor use a general approach (one that is thought to apply to *all* children) when raising and engaging in discussions about death and dying related to childhood. In addition, and as previously highlighted, the relationality of childhood, as it is understood by sociology of childhood, has the consequence of encouraging us to acknowledge that children are influenced by the social context they exist within. As a result, there are a variety of ways that death can be understood and perceived by a child, in virtue of the diverse sociocultural contexts a family and child can exist within. Therefore, to make the assumption that all children before a certain stage cannot talk about death and dying in a meaningful way is to overlook the unique experiences and capacities a particular child may have, informed by their sociocultural position. It is also a reflection of the societal avoidance to adapt conversations to meet the child “where they are at,” and the frequent result of this avoidance is to entirely abandon a conversation or topic. There are definite exceptions to this phenomenon, and many clinicians who are already utilizing non-essentialist approaches to tailor conversations for the young person and their family, but widespread avoidance of these discussions at individual, research, and societal levels still exists and has downstream impacts.

Evidence and normative arguments have emerged indicating the harms young people with illnesses face in the context of silenced death and dying dialogue. For one, young people are entitled to have their views taken seriously in matters that impact their lives (United Nations, 1989) and imposing silences in relation to death (whether with good intent or not) ignores those rights. Most young people want to be fully informed about all aspects of their illnesses (Ellis & Leventhal, 1993) and have their rights realized. Avoiding these conversations has also been a trigger for new issues to arise, such as a “profound sense of loss of control” that is associated with not being informed and perpetuated uncertainties about the future (Beale et al., 2005, p. 3629). This aligns with literature emerging from the psychosocial field regarding disenfranchised grief, or a form of grief that arises by those who experience a loss but cannot openly acknowledge, publicly mourn, or receive social supports for that loss (Corr, 1999; Doka, 1999). As a result, it is possible to presume that for some young people who are left out of conversations pertaining to the end of their life, the grief associated with their possible loss of life may be coupled with grief from not being able to talk about this loss in meaningful ways with many individuals. As such, being involved in discussions about death can have a positive impact on the young person. Furthermore, it can also positively impact families (Aldridge et al., 2017). In one study that examined whether and how death was talked about in/with children who had severe malignant disease, parents who *did* talk about death with their child did not voice regret, but 27% of parents who *did not* talk about death with their critically ill child regretted not having done so (i.e., they wish they had talked about it with their child, before their child passed away) (Kreicbergs et al., 2004). In this way, silenced narratives about death not only impact the young people experiencing an illness, but it also impacts their social circle.

## MAID & Silenced Narratives of Death

Considering the existence of silenced death narratives in the particular context of Canada where Medical Assistance in Dying (MAID) is legal, it becomes possible to imagine that MAID can actually act as a bridge to rectify the perceived incompatibility of hope and death, for both adults and for children. Although MAID is not presently legal for those under the age of 18 in Canada, it seems that the practice of MAID can traverse the divide between hope and death by prioritizing and ensuring the presence of dignity, respect, voice, and a new vision of hope in discussions that pertain to dying and death. In the context of childhood death, the silence consumes the possibility of death narratives and discourse due to fear and resistance to losing hope (particularly hope *for* a cure), and this also prevents discussions about MAID from being seen as relevant because they are seen as a threat to the “hope as cure” paradigm. However, if we recall the motivations that are most prominent for adults when seeking MAID, and the rationale that has been evident through initiatives like “Dying with Dignity,” we remember that loss of autonomy and loss of dignity are amongst the top three most prominent end-of-life concerns and the concerns that lead individuals to consider MAID (Oregon Public Health, 2014; Wiebe et al., 2018). Therefore, MAID as a practice and policy allows for a shift to new understandings of hope to be present in discussions (those that may be theoretical, spiritual, clinical, and research-based in nature) about death.

When we acknowledge and recognize that accessing MAID can or may be (for some, at least) a way to reduce fear of the unknown or a loss of control and dignity at the end of an incurable illness, then the pre-emptive discussions with young people *about MAID* become one way to displace the silence and to claim a voice to speak about childhood death. And, these types of conversations are not solely valuable for those currently facing an incurable or a critical illness, but also for otherwise “healthy” young people who are building their agential capacities and finding the place to share their voice in an adult-driven society. Our ongoing research on MAID centers this idea to make space for such conversations in the Ontario context, and recognizes the need for similar investigations to take place in other jurisdictions.

## Conclusion and Key-Takeaways

The unspeakable nature of death and dying during childhood is a phenomenon marked with conceptual and practical limitations. While this silence can lead to poor quality end-of-life experiences for some young people facing critical illnesses, the literature has also not sufficiently addressed the concept of “unspeakability” to advance conversations and inform relevant clinician training. In this paper, we have highlighted the ways in which the silence associated with child mortality and experiences of death is manifested. We have also explored how both dominant philosophical and sociological conceptions of death and dying, along with rising life expectancy for children and youth (in many countries in the Global North, though not all in the Global South), may contribute to the persistent nature of these silences. Since hope and death arise as binaries, our work has attempted to resist this opposition and find spaces (for example, through MAID) where hope can be maintained at the end-of-life and where conversations about childhood death and dying, both clinically and in research, can exist. Overall, this work offers initial reflections necessary to develop a theoretical framework for dialogue on the unspeakable nature of childhood death and dying.

As highlighted earlier in this piece, many countries face child mortality rates that are significantly higher than the rates in Canada. As such, this paper highlights the need for future empirical inquiries aimed at understanding the potential mediating role of childhood mortality rates in silencing, through: (a) historical document review, to understand whether any historical literature has pertained to conceptualizations of childhood death and dying and degree of associated openness; and (b) developing anthological, cross-cultural studies to explore whether the unspeakable phenomenon is apparent in other countries around the world that face higher child mortality rates.

Moving forward, the tendency in developmental psychology to treat all young people as a uniform group needs to be challenged through critical approaches that recognize the capacity of some children to engage in death-related conversations. These approaches will advance forms of health, social, and psychological care that are attuned to the needs of each individual and family and responsive to the possibility that young people want to be engaged in these hard conversations in meaningful ways. This work also requires scholars, clinicians, and policymakers to avoid Langston Hughes’ proclamation of leaving death as “a note unsaid,” to ensure dialogue on childhood death and dying fills the conceptual and clinical silences.

## Notes

1. Though it is important to note that for some low-income countries, under-five mortality rates are significantly higher than the same rates in high-income countries. The context of the health system, along with other social determinants of death, play a large role for this indicator.

2. Medical assistance in dying (MAID), otherwise referred to as euthanasia, became legal in Canada in 2016 following the *Carter v Canada* Supreme Court decision. MAID refers to the process of having medical intervention to bring about one’s death. In the context of this research, “mature minors” are individuals under the age of 18 who are deemed legally capable of making their own medical decisions.

3. It is important to note that some have claimed that in previous eras where mortality rates were high, death was a natural part of conversation and many spent a considerable amount of time talking and thinking about death and dying (Milner, 1972).
